# Crosstalk of disulfidptosis-related subtypes, establishment of a prognostic signature and immune infiltration characteristics in bladder cancer based on a machine learning survival framework

**DOI:** 10.3389/fendo.2023.1180404

**Published:** 2023-04-19

**Authors:** Songyun Zhao, Lanyu Wang, Wei Ding, Bicheng Ye, Chao Cheng, Jianfeng Shao, Jinhui Liu, Hongyi Zhou

**Affiliations:** ^1^ Department of Urology, Wuxi People's Hospital Affiliated to Nanjing Medical University, Wuxi, China; ^2^ Department of Neurosurgery, Wuxi People's Hospital Affiliated to Nanjing Medical University, Wuxi, China; ^3^ Department of Nuclear Medicine, The First Affiliated Hospital of Nanjing Medical University, Nanjing, China; ^4^ School of Clinical Medicine, Yangzhou Polytechnic College, Yangzhou, China; ^5^ Department of Gynecology, The First Affiliated Hospital of Nanjing Medical University, Nanjing, China

**Keywords:** disulfidptosis, BLCA, machine learning, tumor microenvironment, immunotherapy, risk score signature

## Abstract

**Background:**

Bladder cancer (BLCA) is the most common malignancy of the urinary tract. On the other hand, disulfidptosis, a mechanism of disulfide stress-induced cell death, is closely associated with tumorigenesis and progression. Here, we investigated the impact of disulfidptosis-related genes (DRGs) on the prognosis of BLCA, identified various DRG clusters, and developed a risk model to assess patient prognosis, immunological profile, and treatment response.

**Methods:**

The expression and mutational characteristics of four DRGs were first analyzed in bulk RNA-Seq and single-cell RNA sequencing data, IHC staining identified the role of DRGs in BLCA progression, and two DRG clusters were identified by consensus clustering. Using the differentially expressed genes (DEGs) from these two clusters, we transformed ten machine learning algorithms into more than 80 combinations and finally selected the best algorithm to construct a disulfidptosis-related prognostic signature (DRPS). We based this selection on the mean C-index of three BLCA cohorts. Furthermore, we explored the differences in clinical characteristics, mutational landscape, immune cell infiltration, and predicted efficacy of immunotherapy between high and low-risk groups. To visually depict the clinical value of DRPS, we employed nomograms. Additionally, we verified whether DRPS predicts response to immunotherapy in BLCA patients by utilizing the Tumour Immune Dysfunction and Rejection (TIDE) and IMvigor 210 cohorts.

**Results:**

In the integrated cohort, we identified several DRG clusters and DRG gene clusters that differed significantly in overall survival (OS) and tumor microenvironment. After the integration of clinicopathological features, DRPS showed robust predictive power. Based on the median risk score associated with disulfidptosis, BLCA patients were divided into low-risk (LR) and high-risk (HR) groups, with patients in the LR group having a better prognosis, a higher tumor mutational load and being more sensitive to immunotherapy and chemotherapy.

**Conclusion:**

Our study, therefore, provides a valuable tool to further guide clinical management and tailor the treatment of BLCA patients, offering new insights into individualized treatment.

## Introduction

Bladder cancer is reported to be the most common malignancy of the urinary tract (1). About 900,000 new cases of bladder cancer are diagnosed each year, of which approximately 25% are muscle-invasive bladder cancer and 75% are non-muscle-invasive bladder cancer (2). Radical cystectomy (RC), coupled with cisplatin-based adjuvant chemotherapy, remains the main option of the treatment for muscle-invasive bladder cancer (3). Despite the use of multiple treatment methods, such as surgery, chemotherapy, and radiotherapy, approximately half of bladder cancer patients who undergo radical cystectomy will experience recurrence or metastasis. Therefore, there is a pressing need to develop dependable and efficient prognostic biomarkers that can identify various subgroups of bladder cancer patients, and guide individualized and optimal treatments. This is particularly crucial given the rapid advances in bioinformatics.

Ferroptosis, a form of cell death triggered by the accumulation of iron-dependent lipid peroxides, has gained significant attention in tumor research in recent years. The core mechanism of ferroptosis is the dysregulation of iron ion transport and metabolism within the cell, resulting in the accumulation of excessive iron ions in cells, which subsequently activates lipid peroxidation and inhibits antioxidant reactions, ultimately leading to cell death. The association of ferroptosis with tumor cell proliferation, immunotherapy, and other aspects has been extensively studied, and related research findings have been applied to various aspects of tumor treatment (4–6). A recent study found that cells with high expression of SLC7A11 can inhibit ferroptosis under glucose deprivation conditions through cystine uptake mediated by SLC7A11, but this may induce a new form of cell death, disulfidptosis (7, 8). Current research suggests that the occurrence of disulfidptosis is related to changes in the cellular redox state, and it can promote tumor cell death by altering the conformation of cytoskeletal proteins. Therefore, disulfidptosis may become a new field in tumor treatment, but further research and exploration are needed to understand its specific mechanisms and therapeutic applications.

In recent years, a strong link has been established between disulfide metabolism and cancer. Disulfide metabolism refers to the redox reactions within cells, in which the formation and breakage of disulfide bonds play a major role. Recent studies have shown that many cancer cells experience oxidative stress, leading to disulfide metabolism disorders that affect cancer cell survival and proliferation (9, 10). Additionally, disulfide metabolism in cancer cells is also associated with biological behaviors such as drug resistance, metastasis, and immune escape (11, 12). As a new type of programmed cell death (PCD), disulfidptosis may have a certain correlation with tumor immune response. The cell death signal produced by disulfidptosis may be recognized by tumor immune cells, thereby activating the immune response of tumor-specific T cells and enhancing the effectiveness of humoral and cellular immunity, ultimately improving cancer treatment efficacy. However, more disulfidptosis-related biomarkers need to be established, and further links need to be made between targets and pathways dependent on disulfide metabolism with cancer susceptibility.

Therefore, identifying different clustering features and establishing disulfidptosis-related signatures may effectively predict prognosis and response to immunotherapy in bladder cancer patients. In this study, 758 bladder cancer samples were obtained from the TCGA, GEO, and IMvigor210 databases. The expression levels and mutation status of four DRGs in BLCA were analyzed, and BLCA samples were classified into different clusters according to the expression levels of the DRGs. Subsequently, to explore the impact of different disulfidptosis patterns on patient prognosis, this study constructed a disulfidptosis- related prognostic signature (DRPS), with better immunotherapy outcomes for patients in the low-risk group.

## Materials and methods

### Data collection and procession

RNA-seq data and clinical information for BLCA patients were collected from two sources: GSE 13507 (http://www.ncbi.nlm.nih.gov/geo/) and TCGA-BLCA (https://www.example.com). Additionally, data on transcriptional profiles and clinical characteristics of metastatic BLCA patients treated with anti-PD-L1 immunotherapy were obtained using the “IMvigor 210 CoreBiologies” package (13). FPKM values were converted to transcripts per kilobase million (TPM),and TPM was considered equivalent to transcripts from the GEO microarray (14). Gene expression profiles were measured using the transcript per million estimation and log2-based transformation. The mRNA expressions of BLCA patients from these three cohorts were combined and batch corrected using the “sva” package (15). Four genes that suppress disulfidptosis, namely SLC7A11, SLC3A2, RPN1, and NCKAP1, were obtained from a recent publication (8). These four genes are known as disulfidptosis-related genes (DRGs).

We also downloaded single-cell sequencing data containing 25 untreated muscle-infiltrating bladder cancers from GSE169379 (16). The data were analyzed according to the post-quality control given and the cells were annotated according to the data in the original study. The “FindAllMarkers” and “FindMarkers” functions are used to perform Wilcoxon tests between pairs of cell clusters to find genes specifically expressed in each cluster.The ‘featureplot’ function is used to show the expression of a specific gene.

### Sample source and immunohistochemistry

Tissue samples were obtained from BLCA patients who underwent transurethral resection of bladder tumor (TURBT) or radical cystectomy at Wuxi People’s Hospital affiliated with Nanjing Medical University. All patients signed an informed consent form before the use of clinical material. The study was approved by the Ethics Committee of Wuxi People’s Hospital, Nanjing Medical University (KYLLH2018019).

Immunohistochemistry was performed as follows: paraffin sections were placed in an incubator at 60°C for 4 hours and then defatted in xylene three times for 10 minutes each. The sections were rehydrated through 100%, 90%, 80%, and 70% alcohol. After blocking the endogenous peroxidase activity with 3% hydrogen peroxide for 10 minutes, the slides were rinsed with PBS and antigen retrieval was performed in sodium citrate solution using the autoclaving method. Slides were incubated in blocking buffer for 1 hr to block non-specific antibody binding, then either SLC3A2 antibody (ab307587, 1:5000, Abcam) or RPN1 antibody (ab198508, 1:500, Abcam) was applied overnight and incubated with secondary antibody for a further 1 hr. Color development was performed on slides with DAB substrate before counterstaining with hematoxylin. Finally, positive staining on slides was assessed by microscopy.

### Consensus unsupervised clustering

We performed an unsupervised cluster analysis of the four DRG expressions using the “ConsensusClusterPlus” package to identify different DRG-related clusters (17). The “K-Means” algorithm was applied and “euclidean” was used as a measure of distance, accompanied by resampling of 80% of the items and 1000 replications (18). The optimal k value was determined according to the proportion of ambiguous clustering (PAC). The “limma” R package was used to explore differentially expressed genes (DEG) between two DRG clusters, using |log2 FC| > 1 and FDR< 0.05 as cut-off values (19).

### Establishment of TEX-related prognostic signature in ovarian cancer

We used a novel computational framework that combined machine learning algorithms to identify a disulfidptosis-related prognostic signature (DRPS) by analyzing the expression profiles of the TCGA-BLCA, GSE 13507, and IMvigor 210 cohorts. Differential genes between DRG clusters were obtained through univariate Cox regression analysis, and we used 10-fold cross-validation to test 84 combinations of 10 machine learning algorithms, including Lasso, Ridge, stepwise Cox, CoxBoost, stochastic survival forest (RSF), elastic network (Enet), partial least squares regression for Cox (plsRcox), supervised principal components (SuperPC), generalized boosted regression modeling (GBM), and survival support vector machine (survival-SVM).

To create the disulfidptosis-related prognostic signature (DRPS), a combination of machine learning algorithms including RSF and CoxBoost was used. CoxBoost was employed to filter the most valuable genes, and RSF was then used to derive the most trustworthy models. The RSF algorithm was further used to filter the most reliable model. To split the survival trees, a log-rank score test was conducted, as previously described (20). Firstly, the x-variable was ordered as x1 ≤ x2 ≤ … ≤ xn, and then the “ranks” for each survival time Tj (j ∈ [1, …, n]) were computed using the following equation:


$${\text{a}}_{\text{j}}=\delta_{\text{j}}-\sum_{k=1}^{\Gamma_{j}}\frac{\delta_{k}}{n-\Gamma_{k}+1}$$

where Γj represents the index of the order for Tj and Γk = #[t: Tt ≤ Tk]. The log-rank score test was performed as follows:


$$\text{score}=\text{S}(\text{x},\text{ c})=\frac{\sum_{xk\leq c}(a_{j}-n\iota \bar{a}}{\sqrt{n\iota \left(1-\frac{n\iota }{n}\right)\frac{2}{S_{A}}}}$$

Where $s_{A}^{2}$ and $\bar{a}$ represent the sample variance of [aj: j = 1,…, n] and sample mean, respectively. The log-rank score splitting by | S (x, c) | was used to determine the measure of node separation. The best split is reached by maximizing this value over x and c.

To determine whether DRPS could be an independent prognostic factor for patients with BLCA, univariate and multifactorial Cox regression analyses were conducted in the TCGA and IMvigor 210 cohorts. A nomogram was created using the ‘rms’R package based on age, grade, stage, and risk grouping in the TCGA cohort to predict OS in clinical patients at 1, 3, and 5 years (21).

### Correlation enrichment analysis

We performed Gene Ontology (GO) and Kyoto Encyclopedia of Genes and Genomes (KEGG) analyses using the R package “clusterProfiler” and visualized them with the “circlize” R package. p-values<0.05 were considered significantly enrichment (22, 23). To identify DRG-related differences in biological function, the MsigDB database “c2.cp.kegg.v6.2.symbols.gmts” was used for genomic variation analysis (GSVA) (24). The GSEA (http://software.broadinstitute.org/gsea/index.jsp) was conducted between the two groups to investigate differences in biological function between high and low-risk populations.

### Somatic mutation analysis

Somatic variant data were stored in mutation annotation format (MAF) and we used maftools to analyze mutation data from BLCA samples (25). We calculated the tumor mutation burden (TMB) score for each BLCA patient and explored the relationship between the risk score and TMB. The TMB score was calculated as follows: (total mutations/total covered bases) × 10^6^. The prognostic value of TMB in BLCA was investigated using Kaplan-Meier analysis in the R package (26, 27).

### Tumour microenvironment and immune-related pathways

Based on the expression profile, the R package “estimate” was used to infer the interstitial and immune cell abundance and tumor purity of malignant tumor tissue (28, 29). The tumour-infiltrating immune cells dataset is available for download at TIMER 2.0 (http://timer.cistrome.org). The results of TIMER, CIBERSORT, quantTIseq, MCP-counter, xCELL and EPIC algorithms were also compared and the “ComplexHeatmap” R package was used for further description. To assess immune cell infiltration in BLCA patients with different survival outcomes, we employed single gene set enrichment analysis (ssGSEA) to score 28 immune cell types within the tumor microenvironment (TME) of low-risk (LR) and high-risk (HR) groups. To investigate the differences in biological functions between high and low risk populations, GSEA (http://software.broadinstitute.org/gsea/index.jsp) was performed between the two groups, which was based on the MsigDB database “c2.cp.kegg.v6.2. symbols.gmts” file in the MsigDB database. The threshold values were set at P< 0.05 and FDR< 0.25.

### Predictive methods for immunotherapy and chemotherapy

TIDE (http://tide.dfci.harvard.edu/) represents tumor immune dysfunction and exclusion. It is a computational framework for assessing the potential for tumor immune escape in the gene expression profile of tumor samples (30). To exploit the full potential of this feature in targeted chemotherapy, we used “prophictic” to calculate the IC50 values of four classical chemotherapeutic agents that are effective in treating BLCA. The IC50 values of Doxorubicin, Gemcitabine, Lapatinib, and Sunitinib were compared between high and low-risk groups to test the sensitivity of patients to each drug (31, 32).

### Statistical analyses

We employed various statistical methods to analyze the data, including Kaplan-Meier survival analysis and log-rank tests to determine prognostic values and compare patient survival across different subgroups within each data set. For normally distributed groups, we used the Student’s t-test, and for non-normally distributed variables, we used the Wilcoxon test. To compare multiple groups, we used the Kruskal-Wallis test as a non-parametric method. Correlations between variables were assessed using Spearman’s correlation analysis. We considered a P-value less than 0.05 as statistically significant for all statistical investigations.

## Results

### Expression and mutation of disulfidptosis-related genes in BLCA

**Figure 1:**
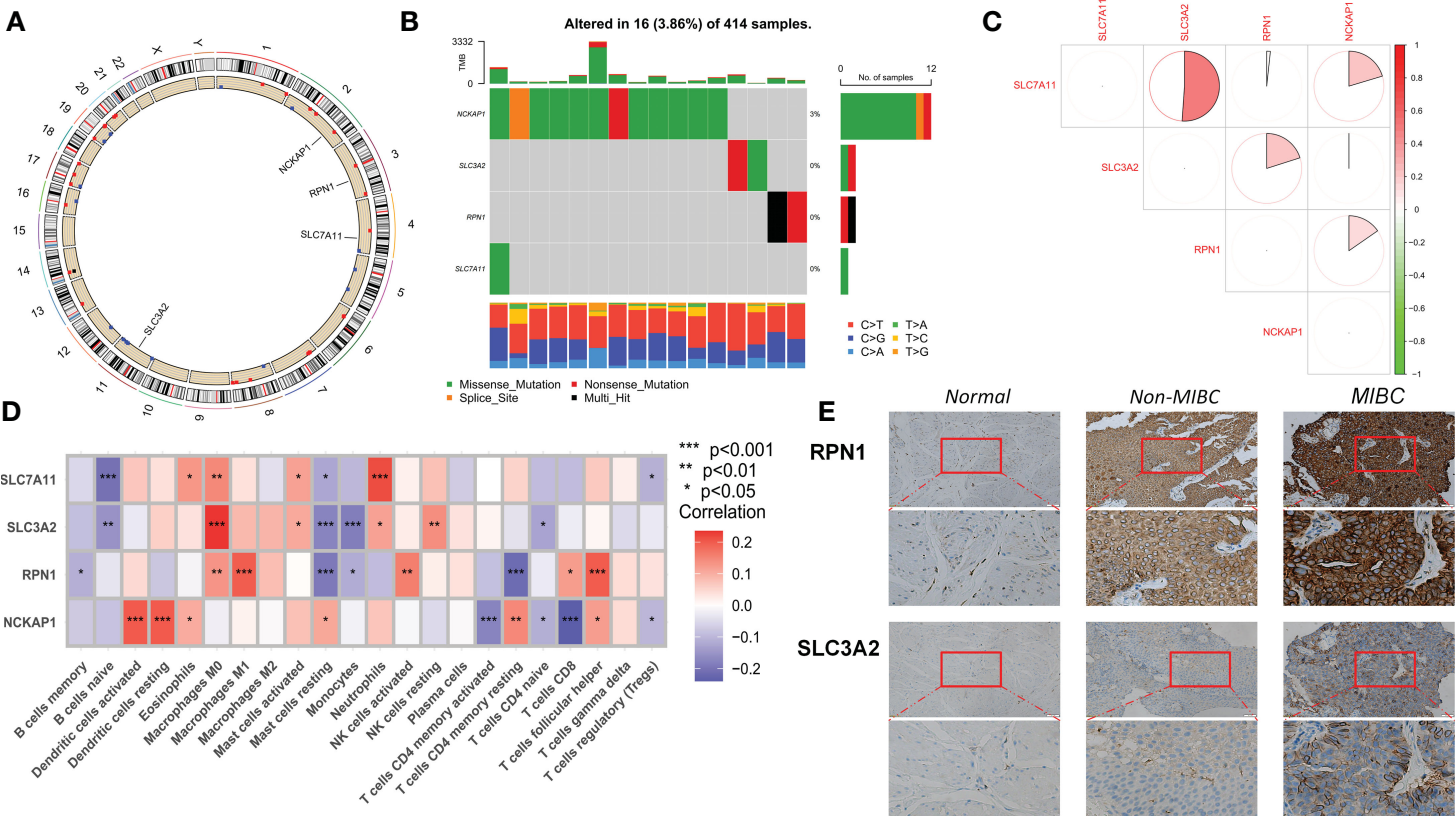
Transcriptional alterations and expression of DRG in BLCA. **(A)** Location of CNV alterations in DRG on chromosome 23. **(B)** Frequency of mutations in the 4 DRGs in 414 BLCA patients from the TCGA cohort. **(C)** Correlation of 4 DRG expressions. **(D)** Correlation of cibersort results showing expression of the 4 DRGs with immune cell infiltration. **(E)** IHC staining of SLC3A2 and RPN1 expression in normal tissue, Non-MIBC, and MIBC samples (at 20× magnification, scale bar, 50μm). MIBC Muscle invasive bladder cancer; *p<0.05; **p<0.01; ***p<0.001.

Of the four DRGs included in this study, a summary analysis of somatic mutations in the TCGA-BLCA cohort showed an extremely low incidence of mutations in the DRGs (**Figure 1B**). In the TCGA cohort, only 16 of the 414 samples (3.86%) had significant DRG alterations, and no alterations were observed in any of the DRGs except NCKAP1. **Figure 1A** shows the localization of the four DRGs on the human chromosome. Spearman correlation analysis showed that the expression of the four DRGs showed a positive correlation overall, with a higher correlation for SLC7A11 and SLC3A2 (**Figure 1C**). According to the results of cibersort analysis, DRGs were strongly associated with the expression of tumor-infiltrating immune cells (TIICs), for example, SLC7A11 and SLC3A2 may be intrinsically associated with macrophages (**Figure 1D**). The results of IHC staining showed that RPN1 and SCL3A2 stained more deeply in tumor tissue, particularly in myxoid-infiltrating tumors (**Figure 1E**). Finally, we merged the TCGA-BLCA, iMvigor210, and GSE13507 cohorts by the “sva “R package to eliminate batch effects between cohorts and perform the next step of the analysis.

**Figure 2:**
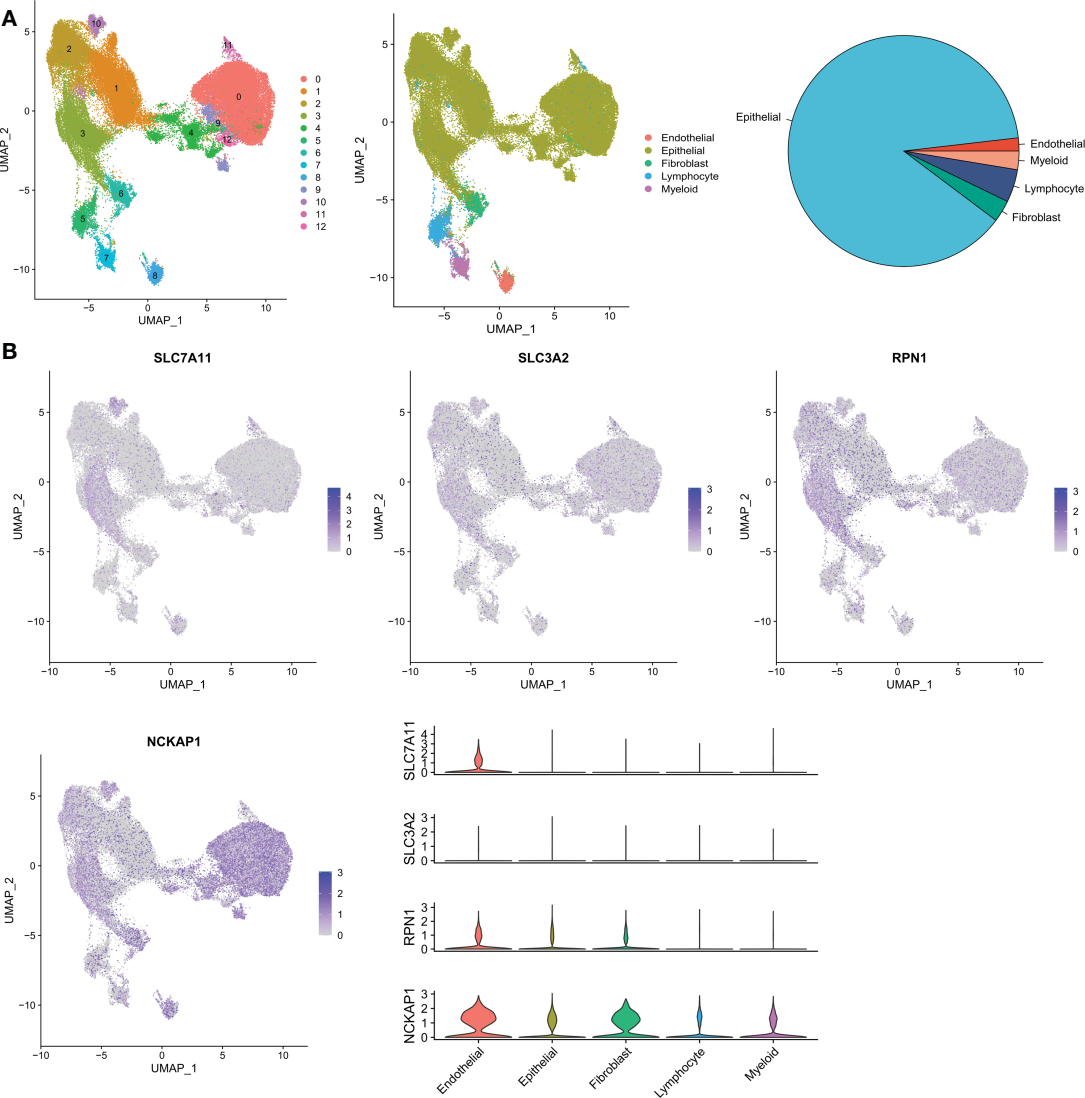
Validation of single-cell RNA sequencing. **(A)** Annotation of all cell types in GSE169379 and the percentage of each cell type. **(B)** Expression of SLC7A11, SLC3A2, RPN1, and NCKAP1 in each cell type.

We used the bladder cancer single-cell dataset GSE169379 to analyze the expression of four DRGs in the tumor microenvironment (TME). There are 13 cell populations and 5 major cell types in the GSE169379 dataset, including endothelial, epithelial, fibroblast, myeloid, and lymphocytes. The majority of these were epithelial cells (tumor cells), and the distribution and numbers of the various cell types are shown in **Figure 2A**. SLC3A2 expression was low in all cells, while NCKAP1 was expressed in all cells to some extent (**Figure 2B**).

### Identification of DRG clusters in BLCA

**Figure 3:**
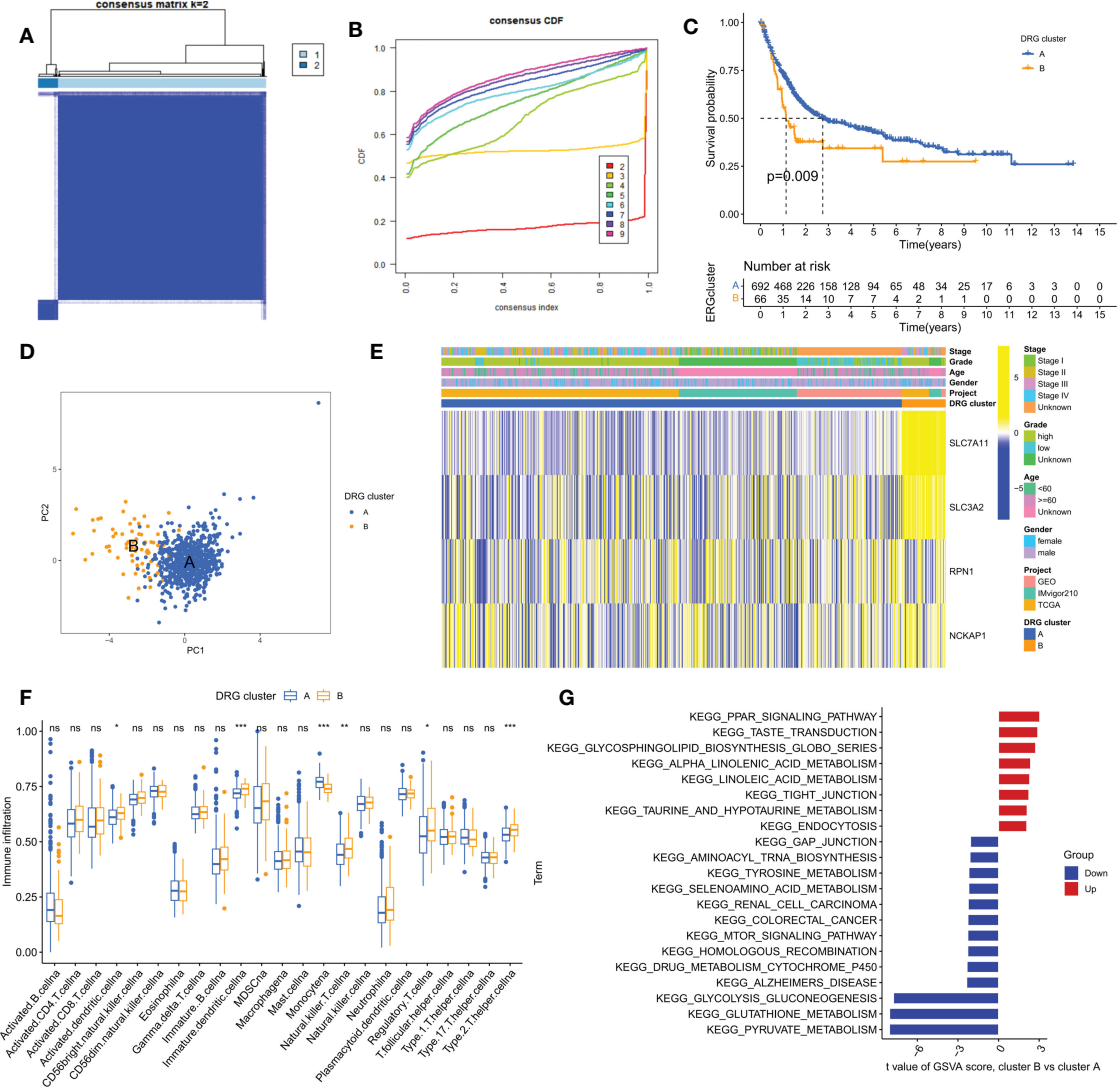
Clinical, pathological, and biological characteristics of two DRG clusters. **(A)** All samples from the TCGA-BLCA, iMvigor210, and GSE13507 cohorts were divided into two clusters using a consensus clustering algorithm (k=2). **(B)** Cumulative Distribution Function (CDF) from k=2 to 9. **(C)** Kaplan-Meier curve shows different overall survival (OS) between the two DRG clusters. **(D)** Principal Component Analysis (PCA) shows significant differences between the two DRG clusters. **(E)** Heatmap shows differences in clinical information and DRG expression between the two DRG clusters. **(F)** Abundance of 23 infiltrating immune cells in the two DRG clusters. **(G)** GSVA of biological pathways between the two DRG clusters. ns, no significance,*p< 0.05, **p< 0.01, ***p< 0.001.

To gain a comprehensive understanding of the expression pattern of disulfidptosis in bladder cancer, we integrated samples from TCGA-BLCA, iMvigor210, and GSE13507 cohorts. Using a consensus clustering algorithm based on the expression of 4 DRGs, we identified two distinct molecular subtypes among 758 BLCA samples (**Figure 3A**; **Table S1**). The optimal number of clusters was determined to be two, as indicated by the lower slope of the Cumulative Distribution Function (CDF) curve (**Figure 3B**). Thus, the integrated cohort was divided into two DRG clusters, with Cluster A exhibiting a significant survival advantage (**Figure 3C**). Principal Component Analysis (PCA) also confirmed significant differences in the distribution of the two DRG clusters (**Figure 3D**). Furthermore, unsupervised clustering of four DRGs in the combined cohort revealed disulfidptosis-related modification patterns with distinct molecular and clinical features (**Figure 3E**). To investigate the role of DRGs in the tumor immune microenvironment (TME), we used the ssGSEA algorithm to assess the correlation between the two clusters and immune cell subtypes. We observed that the two clusters exhibited different immune cell-infiltrating characteristics (ICICs), with higher levels of dendritic cells, natural killer T cells, regulatory T cells, and helper T cells infiltrating Cluster A, which exhibited pro-inflammatory characteristics (**Figure 3F**). Moreover, Gene Set Variation Analysis (GSVA) revealed significant enrichment of amino acid metabolism and tumor-related pathways in Cluster A (**Figure 3G**). These findings suggest that disulfidptosis plays a crucial role in shaping the TME and that the two molecular subtypes exhibit distinct ICICs and molecular pathways.

### Identification of DRG gene clusters in BLCA

**Figure 4:**
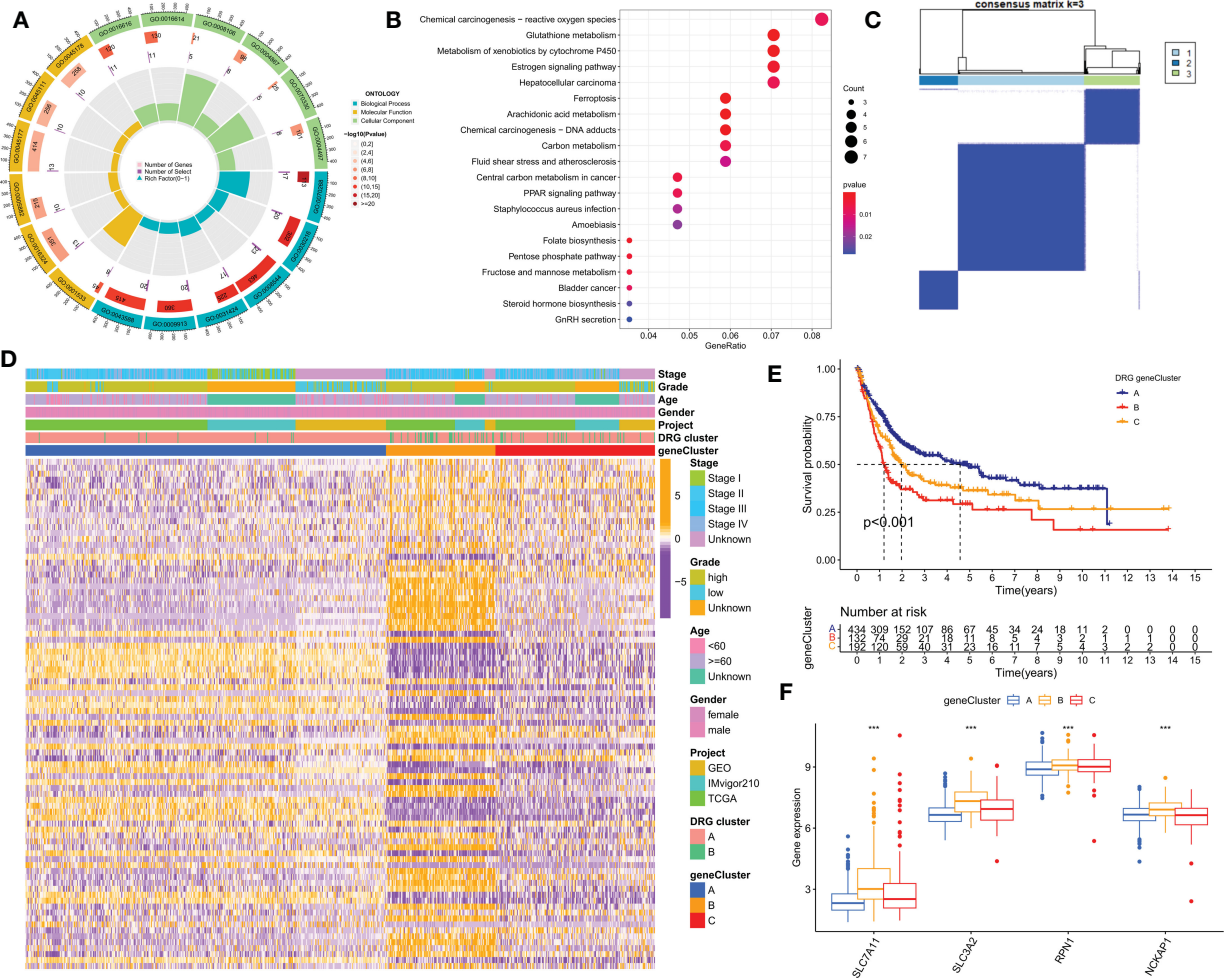
Identification of DRG gene clusters based on differentially expressed genes (DEGs) in DRG clusters. **(A, B)** GO and KEGG enrichment analysis of DEGs in two DRG gene clusters. **(C)** The consensus clustering algorithm (k = 2) was used to divide all samples in TCGA-BLCA, iMvigor210, and GSE13507 cohorts into 3 DRG gene clusters. **(D)** Heatmap of clinical pathological features and expression of DEGs. **(E)** Kaplan-Meier survival analysis of different gene clusters. **(F)** Differences in the expression of four DRGs among different gene clusters. GO, Gene Ontology; KEGG, Kyoto Encyclopedia of Genes and Genomes. ***P< 0.001.

To further investigate the potential biological behaviors of the two disulfidptosis-related subgroups, we identified 153 differentially expressed genes (DEGs) using the “limma” R package. Functional enrichment analysis revealed that these DEGs were mainly enriched in tumor and reactive oxygen species-related signaling pathways (**Figures 4A, B**). We then performed univariate Cox analysis on these DEGs and identified 86 that were significantly associated with prognosis (p<0.05). Next, using the “ConsensusClusterPlus” R package, we divided the BLCA patients into 3 DRG gene clusters (**Figure 4C**; **Table S2**). DRG gene cluster B had a worse prognosis (**Figure 4E**), and the heatmap (**Figure 4D**) showed the expression levels of prognosis-related DEGs and clinical pathological factors in the two DRG clusters and two DRG gene clusters. Furthermore, all four DRGs were expressed higher in DRG gene cluster B, which confirmed the worse clinical outcome in patients in gene cluster B (**Figure 4F**).

### Construction of the disulfidptosis-related prognostic signature

**Figure 5:**
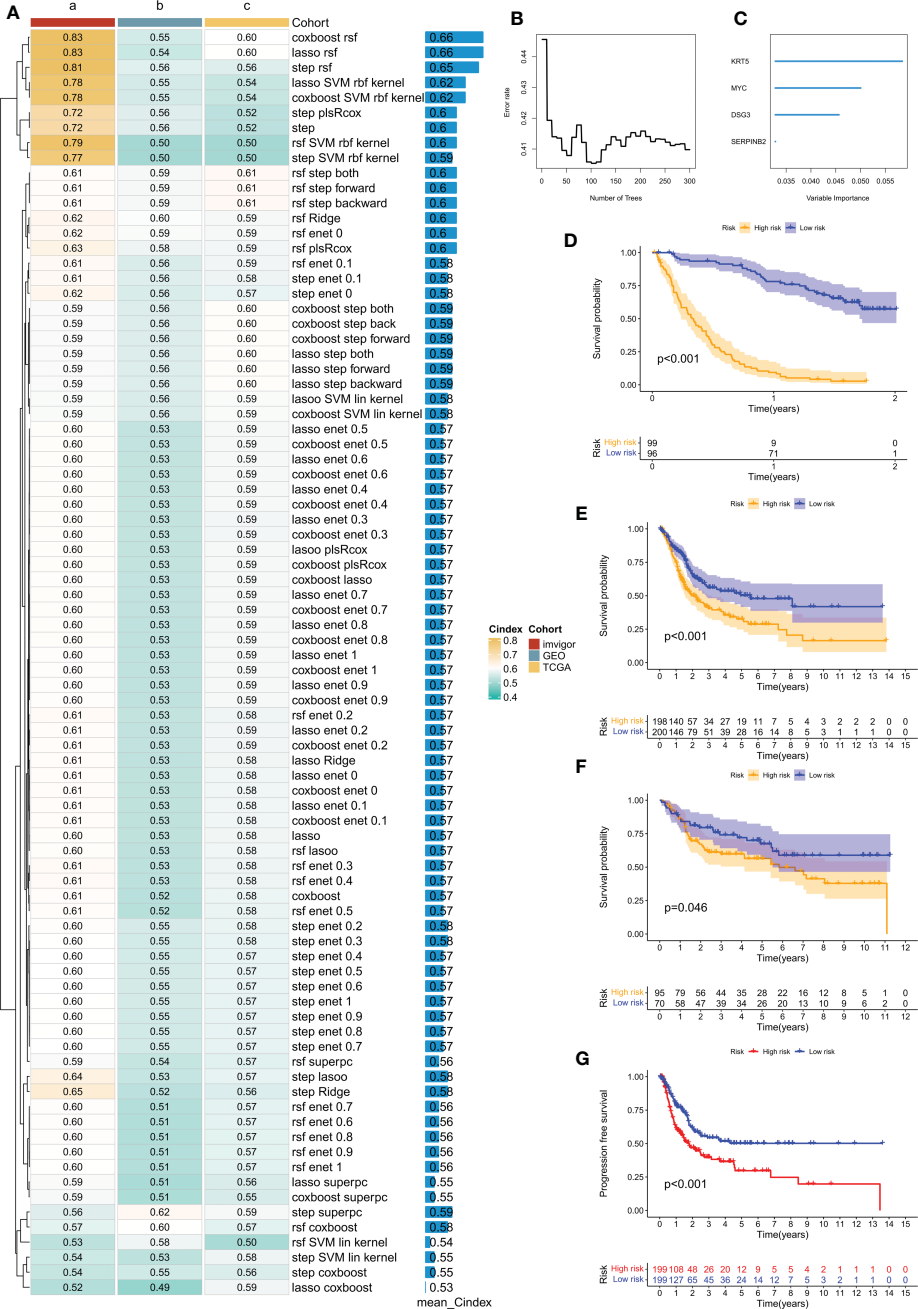
Prognostic features related to disulfidptosis constructed through machine learning-based integration and their prognostic value. **(A)** 84 integrated prediction models based on machine learning were fitted using 10-fold cross-validation. The C-index was computed for each model in both the training and validation cohorts, which included TCGA-BLCA, iMvigor210, and GSE13507 cohorts. **(B)** The number of trees determined by minimum error. **(C)** Importance of the four most valuable genes based on the RSF algorithm. **(D–F)** Kaplan-Meier survival curves of overall survival (OS) for high-risk and low-risk groups of patients in the iMvigor210, TCGA-BLCA, and GSE13507 cohorts, respectively. **(G)** Kaplan-Meier survival curve of progression-free survival (PFS) for high-risk and low-risk groups of patients in the TCGA-BLCA cohort.

The iMvigor210 cohort, which included 86 patients, was used as the training set. Based on the 153 DEGs identified by previous univariate Cox analysis as seed genes, our machine learning-based integrative procedure was used to construct a disulfidptosis-related prognostic signature (DRPS). Ten-fold cross-validation was performed on 84 machine learning-based integrated prediction models in the training set, and the C-index of each model was further calculated in all datasets. Considering that evaluating a model not only depends on its robust performance in the training set but also its performance in the validation set, the best model was the combination of CoxBoost and Random Survival Forest (RSF), which had the highest average C-index (0.66) (**Figure 5A**). Based on the CoxBoost and RSF algorithms, the best-performing DRPS was established, where the CoxBoost algorithm identified four DRG cluster-related genes. Then, RSF was performed to construct the most reliable prognostic model (**Figures 5B, C**). The risk score for each patient was calculated using the formula described in the above method. According to the median risk score in the training set (iMvigor210), the patients in the three cohorts were divided into high-risk (HR) and low-risk (LR) groups. It is worth noting that the LR group had a higher overall survival (OS) rate than the HR group in the iMvigor210 cohort (**Figure 5D**). Furthermore, the prognostic value of DRPS was validated in the TCGA and GEO cohorts by using the same cutoff value. For the TCGA-BLCA and GSE13507 cohorts, patients in the LR group had better OS (**Figures 5E, F**). Similarly, in the TCGA cohort, the PFS of patients in the LR group was better than that of those in the HR group (**Figure 5G**).

### Clinical features and establishment of a prognostic nomogram

**Figure 6:**
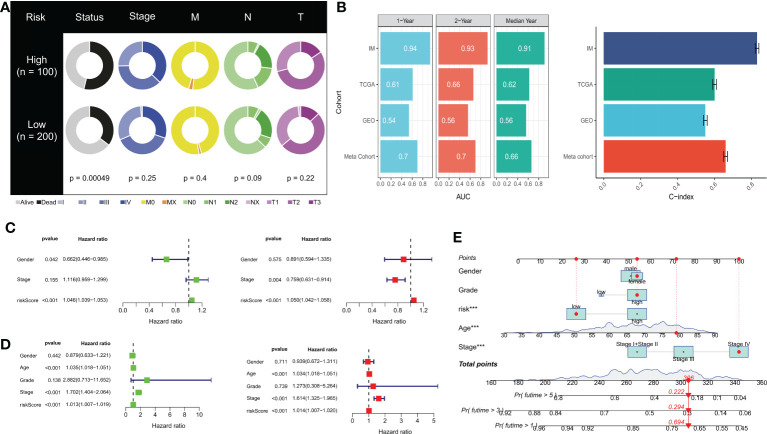
Prognostic value of risk scores and clinical characteristics of BLCA patients. **(A)** Pie chart showing the survival status and clinical stage of patients in high and low-risk groups. **(B)** AUC values for 1-year, 2-year, and median survival time in TCGA-BLCA, iMvigor210, GSE13507, and meta cohorts. Concordance index (C-index) for TCGA-BLCA, iMvigor210, GSE13507, and meta cohorts. **(C)** Univariate and **(D)** multivariate Cox analysis evaluating prognostic and clinical features, including age, grade, and stage. **(E)** Nomogram of risk score and clinical characteristics for predicting 1-, 3-, and 5-year survival rates in the TCGA cohort. ***P< 0.001.

We observed significant differences in survival outcomes between patients in the HR and LR groups, although there were no significant differences in the clinical stage (**Figure 6A**). Our DRPS was effective in predicting the OS of patients in the iMvigor210, TCGA, and meta cohorts, with AUCs above 0.6. However, due to possible batch effects when merging chip data and sequencing data, the AUCs for 1-year, 2-year, and median survival times in the GSE13507 cohort were not ideal. We calculated the mean and variance of the C-index through bootstrap resampling and found that the C-index of the meta-cohort was relatively ideal (**Figure 6B**). In both the iMvigor210 and TCGA cohorts, univariate and multivariate Cox analyses showed that the risk score could serve as an independent prognostic factor for BLCA patients compared with other common clinical features (**Figures 6C, D**). In order to make the risk model clinically applicable and practical, we built a Nomogram based on the TCGA cohort and used age, gender, class and stage as predictors of overall survival status in patients with BLCA (**Figure 6E**). In addition to this, the sample risk scores for DRG cluster B and gene cluster B with poorer prognosis were higher in the consensus clustering analysis (**Supplementary Figure 1**).

### TMB analysis and survival analysis of TMB

**Figure 7:**
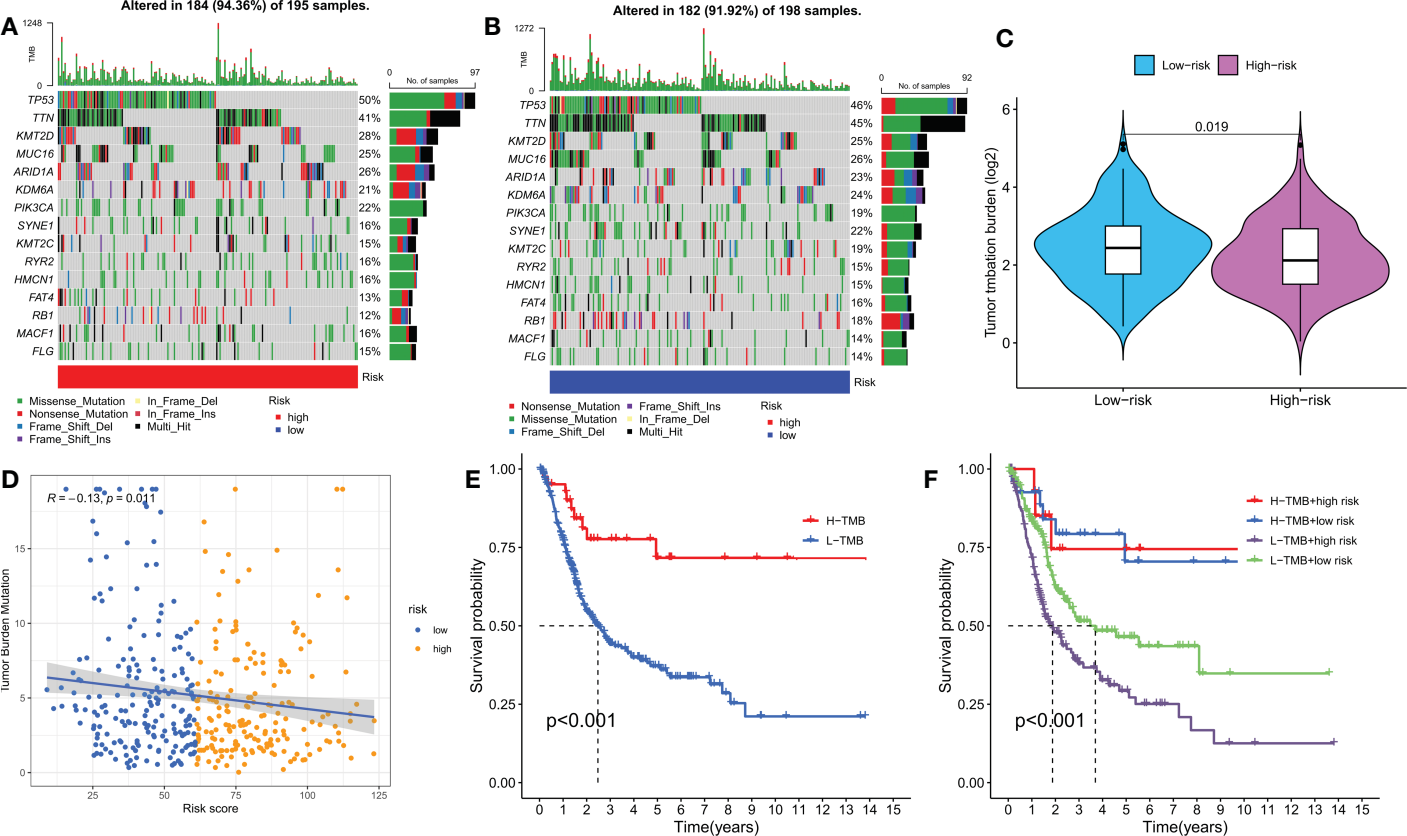
Mutation analysis based on the risk score model. **(A, B)** Waterfall plots summarizing the mutation status of high and low-risk patients. **(C)** The difference in tumor mutation burden between high and low-risk score groups. **(D)** Correlation between risk score and TMB. **(E)** Kaplan-Meier curves of high and low TMB groups. **(F)** Kaplan-Meier curves of four groups classified by risk score and TMB.

Genetic mutations are well-known contributors to the development of tumors. In this study, we examined somatic mutation data based on DRPS in the TCGA database and identified the top three genes with mutations in the HR and LR groups, which were TP53, TTN, and KMT2D (**Figures 7A, B**). It has been reported that different wild-type mutation statuses and expression patterns can lead to different clinical outcomes of immune responses. Therefore, we analyzed tumor mutational burden (TMB) and found a significant difference between the two groups, with the LR group having a higher TMB (**Figure 7C**). Interestingly, we also observed a significant negative correlation between the risk score and TMB (**Figure 7D**). Furthermore, we divided patients into high and low TMB groups based on the median TMB value and performed a K-M survival analysis. The results showed that patients in the high TMB group had a better prognosis, indicating that TMB may be an indicator of poor prognosis in BLCA patients (**Figure 7E**). To further investigate the combined effect of risk score and TMB on patient survival, we divided patients into four subgroups and performed a survival evaluation. The results indicated that the low TMB and HR group had the worst prognosis, which confirmed the effectiveness of our model and identified the best prognostic subgroup for clinical use (**Figure 7F**). In conclusion, our analysis of somatic mutations and TMB further supports the prognostic significance of our DRPS and provides valuable insights into the underlying mechanisms of BLCA development and progression.

### Tumor microenvironment and immune cell infiltration

**Figure 8:**
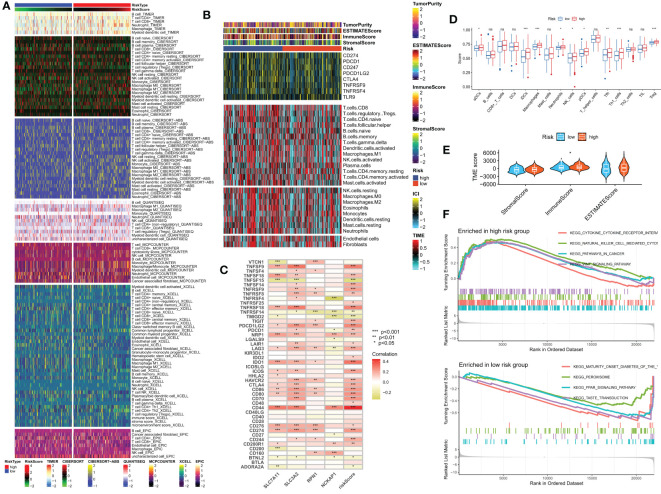
Analysis of the immune microenvironment in different risk groups. **(A)** Differences in immune infiltration status between different risk groups were evaluated by seven algorithms. **(B)** Heatmap showing differences in TME score, immune checkpoint expression, and immune cell infiltration calculated by Cibersort among different risk subgroups. **(C)** Correlation between the expression of all immune checkpoints and DRG and risk scores. **(D)** Differences in immune cell infiltration between different risk groups. **(E)** Differences in TME scores between different risk groups. **(F)** GSEA analysis of different KEGG pathways focused on high and low-risk groups. ns, no significance, *p< 0.05, **p< 0.01, ***p< 0.001.

We investigated the impact of the tumor microenvironment (TME) on clinical outcomes and treatment response in patients, particularly the role of tumor-infiltrating immune cells (TIICs) which significantly affect tumor progression and treatment efficacy (33, 34). Using algorithms from the XCELL, TIMER, QUANTISEQ, MCPCOUNTER, CIBERSORT, CIBERSORT-ABS, and EPIC platforms (**Figure 8A**), we studied the immune landscape of the HR and LR groups. We quantified the enrichment scores of different immune cell subgroups using the “ssGSEA” method to investigate the correlation between risk score and immune cells and their functions. The results showed that the HR group had higher immune cell infiltration scores (**Figure 8D**). We also evaluated the correlation between risk scores and immune checkpoint expression since abnormal expression and function of immune checkpoint molecules have a significant impact on tumor immunotherapy. Most immune checkpoint genes and 4 DRGs showed a strong correlation. Our risk score was positively correlated with ICs expression (**Figure 8C**). Using ESTIMATE, we calculated the proportion of matrix and immune cells in different risk groups to estimate tumor purity (**Figure 8E**) and visualized the ICs, immune scores, and immune cell infiltration of different risk groups using a heatmap (**Figure 8B**). These results suggest that patients in the HR group have poorer prognosis but more active immune function, which may make them more sensitive to immunotherapy. To explore potential differences in biological functions between risk groups, we performed GSEA and identified the four most important enriched signaling pathways. The HR group was mainly associated with tumor and cytokine-related pathways, whereas the LR group was enriched in peroxide-related pathways (**Figure 8F**).

### Immunotherapy and chemotherapy predictions

**Figure 9:**
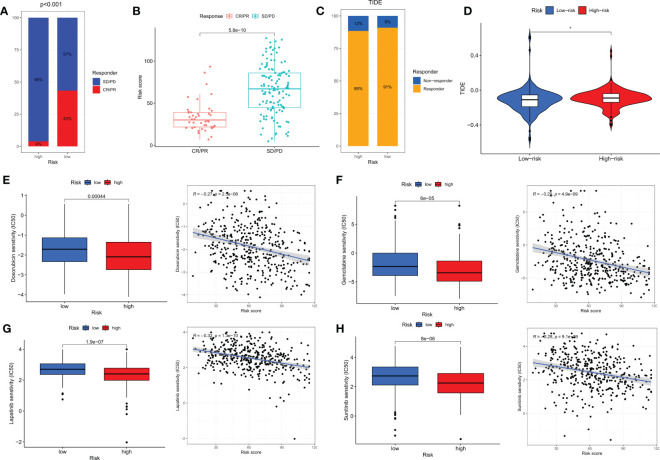
Immunotherapy and chemotherapy in different risk groups. **(A)** Proportions of response to anti-PD-L1 immunotherapy in high- and low-risk groups. **(B)** Differences in risk scores between different response groups in the IMvigor210 cohort. **(C)** Proportions of response to immunotherapy in high- and low-risk groups based on TIDE results. **(D)** Differences in TIDE scores between high- and low-risk groups. **(E–H)** Differences in chemotherapy sensitivity of Doxorubicin, Gemcitabine, Lapatinib, and Sunitinib among different risk groups. CR, complete response; PR, partial response; SD, stable disease; PD, progressive disease. *p<0.05.

Immune checkpoint blockade (ICB) has made significant progress in the treatment of cancer (35, 36). However, ICB treatment is only effective for a subset of patients. To further explore the role of risk scores in immune therapy, we applied the TIDE score to evaluate the potential immune dysfunction in tumors and regional lymph nodes. The results showed that patients in the LR group had a higher probability of response to immune therapy (**Figures 9C, D**). In addition, we also used the IMvigor 210 cohort of anti-PD-L1 immune therapy to predict the response to immune therapy. The risk scores of different response groups showed significant differences, with a higher proportion of CR/PR in the LR group (**Figures 9A, B**). We analyzed the relationship between the risk scores and the IC50 values of four commonly used clinical bladder cancer chemotherapy drugs. We found that Doxorubicin, Gemcitabine, Lapatinib, and Sunitinib were more sensitive in the LR group (**Figures 9E–H**).

## Discussion

Non-muscle invasive bladder cancer (NMIBC) has a higher risk of recurrence and progression to invasive disease due to its high risk of recurrence, particularly high-grade T1 (HGT1) bladder cancer, which accounts for 25% to 43% of all NMIBC cases (37). HGT1 bladder cancer has a recurrence risk of around 40% and a risk of progression to muscle-invasive bladder cancer (MIBC) of approximately 21% (37). Additionally, up to 50% of advanced bladder cancer patients may be ineligible for cisplatin treatment due to renal dysfunction, poor functional status, or comorbidities (38). Immunotherapy has emerged as a potentially revolutionary approach for NMIBC treatment, improving patient survival and offering the possibility of a cure.

In the treatment of bladder cancer, immune checkpoint inhibitors (ICIs) have been proven to be an effective treatment strategy. However, bladder cancer patients have varying responses to ICIs, and resistance exists (39). This necessitates the exploration of new treatment strategies, with disulfidptosis potentially becoming a future research hotspot. A large body of research suggests that treatment targeting disulfidptosis may become a new strategy for bladder cancer treatment. Firstly, the metabolism of disulfides is closely related to the regulation of the redox state, which is one of the important mechanisms of tumor occurrence and development (40). For example, some tumor cells increase their survival and tolerance by altering the intracellular redox environment (41). In addition, some studies have shown that disulfides may have potential application value in tumor treatment. For example, some anti-tumor drugs such as cisplatin and paclitaxel exert their anti-tumor effects by reacting with intracellular disulfides (42, 43). Furthermore, a large body of research has revealed that SLC7A11 drives ferroptosis resistance and plays a regulatory role in tumors and other diseases (44, 45). Therefore, balancing ferroptosis and disulfidptosis may become a new treatment strategy to improve the treatment response rate and survival rate of bladder cancer patients.

BLCA is highly heterogeneous, resulting in variable clinical outcomes and treatment sensitivities. To address this issue, we investigated the potential of disulfidptosis in hindering the progression of BLCA and developed a novel disulfidptosis-related signature to enable risk stratification and personalized treatment prediction. We began by analyzing differential expression levels and genetic mutation characteristics of four DRGs. By applying unsupervised clustering methods to the DRG transcriptome expression levels, we identified two distinct DRG subgroups, namely DRG cluster A and cluster B. Interestingly, compared to patients in cluster B, those in cluster A exhibited better prognosis albeit lower immune infiltration levels. We further identified 86 DEGs between the two DRG subgroups and established two gene clusters based on these DEGs. Significantly, we observed statistically significant OS differences between the two gene subgroups, revealing a close association between gene clusters and DRG clusters. It is noteworthy that traditional modeling articles employing Lasso methods require summarizing the expression levels of multiple target genes into one index and using that index to establish the final model (46–48). The Lasso method is a compressive estimation technique that shrinks variable sets by constructing a penalty function to compress the coefficients of variables and set some regression coefficients to zero, thereby achieving the goal of variable selection. However, the Lasso regression has inherent limitations, such as the inability to obtain explicit solutions, unstable results when using approximations to calculate, and some degree of error (49). To overcome these shortcomings, we transformed 10 machine learning algorithms into over 80 combinations and selected the optimal algorithm based on the average C-index of three BLCA cohorts. This enabled us to construct a stable and powerful disulfidptosis-related prognostic signature (DRPS) for assessing tumor patient prognosis, recurrence and benefits (50). The combination of CoxBoost and RSF was ultimately determined to be the best approach to build a new prognostic model. Our DRPS was found to be an independent prognostic factor for BLCA, and a significant difference in prognosis was observed between the two groups. Furthermore, our nomination graph demonstrated that the disulfidptosis -related prognostic features had a significant advantage over various indicators currently used in clinical practice.

Our study has identified four disulfidptosis suppressor genes, namely SLC7A11, SLC3A2, RPN1, and NCKAP1, which play significant roles in the development and immune infiltration of bladder cancer. SLC7A11, a transmembrane protein, transports extracellular cystine into cells for cysteine production and GSH biosynthesis. SLC7A11 helps counteract oxidative stress and inhibit ferroptosis by maintaining cellular GSH levels. Studies have shown that high expression of SLC7A11 is closely related to cisplatin resistance in bladder cancer (51, 52). Experimental and bioinformatics studies have also demonstrated that the level of SLC3A2 protein is significantly elevated in bladder cancer tumor cells compared to non-cancerous bladder epithelial cells, suggesting that SLC3A2 may serve as a useful tumor tissue marker for the diagnosis of bladder cancer patients (53–55). Regarding RPN1, few studies have shown that inhibiting its expression can help design multiple drug combinations to treat bladder cancer (56). The expression level of NCKAP1 protein is closely associated with the histological tumor grade, metastasis, and low survival rate of various cancer patients (57–59).In bladder cancer (BLCA), the prognostic value of PD-L1 expression is well-established. A recent meta-analysis, which included 11 studies and a total of 1697 BLCA patients, reported a significant correlation between high PD-L1 expression in tumor cells and advanced tumor stage as well as distant metastasis. Consistent with our study, high PD-L1 expression is also associated with poor overall survival (OS) (60). Risk scoring and positive expression of PD-1, PD-L1, and CTLA-4 further support the possibility of immune inhibitory signals in the HR group. High PD-L1 expression can induce an inhibitory immune microenvironment and is associated with poor prognosis in BLCA patients (61, 62). Furthermore, PD-1 can negatively regulate immune responses, which can lead to the initiation of anti-tumor T cells (63). Existing algorithms for predicting immunotherapy response have been successful in predicting the effectiveness of immunotherapy in tumors. The TIDE algorithm is the most popular and widely used prediction tool and was used to explore the immunotherapy value of disulfidptosis-related signatures (64). We found that the HR group had significantly higher TIDE scores and lower effective immune response rates. Results from a real immunotherapy cohort also suggest that patients in the LR group were more suitable for immunotherapy.

According to the results predicted by the chemotherapy drug sensitivity, we found that most chemotherapy drugs, such as Lapatinib and Sunitinib, perform better in the LR group. The effectiveness of Lapatinib and Sunitinib in bladder cancer may be related to their inhibition of ErbB1 or ErbB2 tyrosine kinase activity (65). Doxorubicin is a broad-spectrum anti-tumor drug currently used clinically, with therapeutic effects on various tumors. A study found that the mitochondrial outer membrane protein FUNDC2 promotes cell ferroptosis by regulating the stability of the SLC7A11 protein, thereby participating in the molecular mechanism of Doxorubicin-induced cardiomyopathy (66). Interestingly, we found in our study that patients in the LR group had a higher sensitivity to Doxorubicin, which may further elucidate the biological mechanisms and dynamic balance between ferroptosis and disulfidptosis in tumors.

In this study, we emphasized the significant differences in anti-tumor immune response and immune status among several DRG clusters in BLCA. In addition, we created a new predictive signature related to disulfidptosis, providing new data and findings for bladder cancer biomarkers and their clinical applications. This feature not only accurately predicts the prognosis of BLCA patients, but also brings more benefits to these patients of low-risk groups in terms of prognosis and personalized treatment.

Although our disulfidptosis-related prognostic signature has an outstanding ability to identify patients’ immune landscape and predict their prognosis, some limitations still need to be acknowledged and appropriate methods need to be found to address them in subsequent studies. Data analysis based on public database data may lead to biased prediction results compared to actual situations. Although we have taken some methods to minimize this situation, more data from BLCA patients are needed to verify the practicality of the model and the accuracy of immunotherapy prediction. In addition, more prospective studies and basic research are needed to refine the relevant details of this study.

## Data availability statement

The datasets presented in this study can be found in online repositories. The names of the repository/repositories and accession number(s) can be found in the article/**Supplementary Material**.

## Ethics statement

The studies involving human participants were reviewed and approved by the Ethics Committee of Wuxi People’s Hospital, Nanjing Medical University. The patients/participants provided their written informed consent to participate in this study.

## Author contributions

SZ and LW conceived the study. HZ, WD and SZ drafted the manuscript. BY and CC analyzed and visualized the data. JS, JL and HZ helped with the final revision of this manuscript. All authors contributed to the article and approved the submitted version.

## Supplementary material

Supplementary Table 1 Queue information obtained for the two DRG clusters.

Supplementary Table 2 Detailed cohort information for the 2 DRG gene clusters by the consensus clustering algorithm.

Supplementary Figure 1 Risk scores for different DRG clusers and gene clusters.
