# Supplementary figures and images for: Crosstalk of disulfidptosis-related subtypes, establishment of a prognostic signature and immune infiltration characteristics in bladder cancer based on a machine learning survival framework
Source: Front Endocrinol (Lausanne). 2023 Apr 19;14:1180404. doi: 10.3389/fendo.2023.1180404 (PMC10154596; doi:10.3389/fendo.2023.1180404)

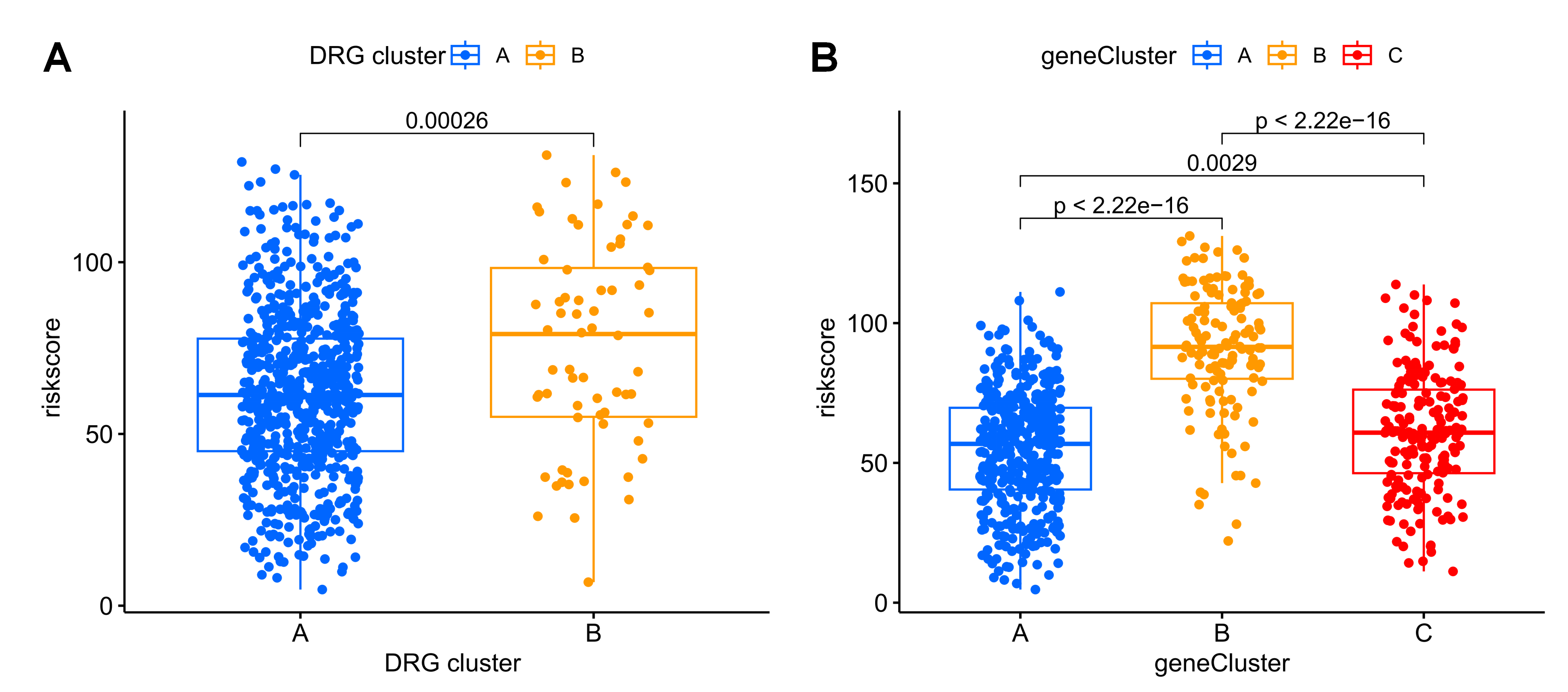

Supplement: Supplementary Figure 1 — Risk scores for different DRG clusers and gene clusters. [file Image_1.tif]
